# Ethane-1,2-diylbis(methyl­phosphinic acid)

**DOI:** 10.1107/S1600536807068122

**Published:** 2008-01-09

**Authors:** Guido J. Reiss, Judith S. Engel

**Affiliations:** a Institut für Anorganische Chemie und Strukturchemie, Lehrstuhl für Material- und Strukturforschung, Heinrich-Heine-Universität Düsseldorf, Universitätsstrasse 1, D-40225 Düsseldorf, Germany

## Abstract

In the title compound, C_4_H_12_O_4_P_2_, there are two crystallographically independent half-mol­ecules in the asymmetric unit, both molecules lying on centres of symmetry. Each mol­ecule is connected on both sides to neighbouring mol­ecules *via* strong O—H⋯O hydrogen bonds. The –POOH groups accept and donate one hydrogen bond in interactions with the neighbouring –POOH group of the adjacent mol­ecule, to give one-dimensional chains along [10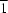]. As each phosphinic acid group donates and accepts one hydrogen bond, the connection between the mol­ecules is best described by a ring motif which can be classified by the Etter symbol *R*_2_^2^(8).

## Related literature

For related literature, see: Bruckmann *et al.* (1999 ▶); Etter *et al.* (1990 ▶); Sicken *et al.* (2000 ▶).
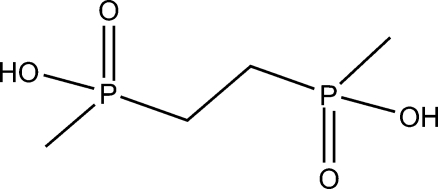


## Experimental

### 

#### Crystal data

C_4_H_12_O_4_P_2_
*M* *_r_* = 186.08
Monoclinic, 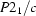

*a* = 6.7761 (18) Å
*b* = 18.703 (8) Å
*c* = 6.8401 (15) Å
β = 102.09 (3)°
*V* = 847.7 (5) Å^3^
*Z* = 4
Mo *K*α radiation
μ = 0.47 mm^−1^
*T* = 290 (2) K
0.40 × 0.35 × 0.30 mm

#### Data collection

Nicolet/Siemens P2_1_/P3 four-circle diffractometer
Absorption correction: none
4847 measured reflections
2466 independent reflections
1879 reflections with *I* > 2σ(*I*)
*R*_int_ = 0.023
3 standard reflections every 100 reflections intensity decay: none

#### Refinement

*R*[*F* ^2^ > 2σ(*F* ^2^)] = 0.035
*wR*(*F* ^2^) = 0.079
*S* = 1.01
2466 reflections
139 parameters
All H-atom parameters refined
Δρ_max_ = 0.35 e Å^−3^
Δρ_min_ = −0.34 e Å^−3^

### 

Data collection: *R3m/V Software* (Siemens, 1989 ▶); cell refinement: *R3m/V Software*; data reduction: *R3m/V Software*; program(s) used to solve structure: *SHELXS97* (Sheldrick, 1997 ▶); program(s) used to refine structure: *SHELXL97* (Sheldrick, 1997 ▶); molecular graphics: *DIAMOND* (Brandenburg, 2001 ▶); software used to prepare material for publication: *SHELXL97*.

## Supplementary Material

Crystal structure: contains datablocks I, global. DOI: 10.1107/S1600536807068122/pk2077sup1.cif

Structure factors: contains datablocks I. DOI: 10.1107/S1600536807068122/pk2077Isup2.hkl

Additional supplementary materials:  crystallographic information; 3D view; checkCIF report

## Figures and Tables

**Table 1:** Hydrogen-bond geometry (Å, °)

*D*—H⋯*A*	*D*—H	H⋯*A*	*D*⋯*A*	*D*—H⋯*A*
O4—H4⋯O2	0.95 (4)	1.55 (4)	2.504 (2)	178 (3)
O1—H1⋯O3	0.94 (4)	1.56 (4)	2.499 (2)	179 (4)

**Fig. 1.**
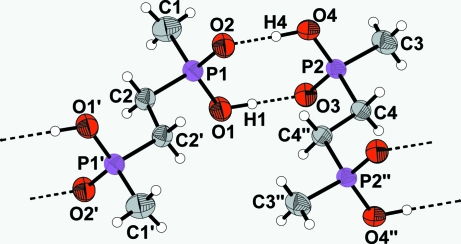
: Part of the hydrogen bonded chain of the title structure. (displacement ellipsoids at the 50% probability level, H-atoms drawn with arbitrary radius, ' = -x, 1 - y, 2 - z; '' = 1 - x, 1 - y, 1 - z)

## supplementary crystallographic information

### Comment

The title compound, [HOO(CH_3_)P-(CH_2_)_2_-P(CH_3_)OOH, crystallizes in the monoclinic centrosymmetric space group *P*2_1_/*c* with two crystallographically independent molecules in the asymmetric unit both of them lying on a centre of symmetry. The molecules are connected on both sides to the next molecules *via* strong O—H···H hydrogen bonds. The bond lengths and angles in the two crystallographic independent molecules are identical within the ranges of their standard uncertainties. As each posphinic acid group donates and accepts one hydrogen bond the motif of this connection between the molecules is best described by an eight-membered ring (Fig.1) which can be classified by the Etter symbol *R*^2^_2_(8) (Etter *et al.*, 1990). A motif which is well known for acetic acid and its derivatives. Each –POOH group accepts and donates one hydrogen bond to the neighbouring –POOH groups of the next molecules to give a one-dimensional chains along [10–1]. This was surprising to us, as the very similar ethane-1,2-diylbis(phosphinic acid) forms a two-dimensional hydrogen bonded network (Bruckmann *et al.*, 1999).

### Experimental

The title compound is generally available by methods described in the literature (Sicken *et al.*, 2000). Recrystallization of the raw material from ethanolic solution at room temperature gave block shaped, colourless crystals.

### Refinement

After refinement of all non-hydrogen atoms using anisotropic displacement parameters, all H atom positions were obtained from successive difference Fourier synthesis. Atom coordinates as well as individual *U*_iso_ values are refined freely for each hydrogen atom.

### Crystal data

**Table:**

C_4_H_12_O_4_P_2_	*F*_000_ = 392
*M**_r_* = 186.08	*D*_x_ = 1.458 Mg m^−^^3^
Monoclinic, *P*2_1_/*c*	Mo *K*α radiation λ = 0.71073 Å
Hall symbol: -P 2ybc	Cell parameters from 37 reflections
*a* = 6.7761 (18) Å	θ = 5.1–14.3º
*b* = 18.703 (8) Å	µ = 0.47 mm^−^^1^
*c* = 6.8401 (15) Å	*T* = 290 (2) K
β = 102.09 (3)º	Block, colourless
*V* = 847.7 (5) Å^3^	0.40 × 0.35 × 0.30 mm
*Z* = 4	

### Data collection

**Table:**

Nicolet/Siemens P21/P3-four-circle diffractometer	*R*_int_ = 0.023
Radiation source: fine-focus sealed tube	θ_max_ = 30.0º
Monochromator: graphite	θ_min_ = 2.2º
*T* = 290(2) K	*h* = 0→9
ω scans	*k* = −26→26
Absorption correction: none	*l* = −9→9
4847 measured reflections	3 standard reflections
2466 independent reflections	every 100 reflections
1879 reflections with *I* > 2σ(*I*)	intensity decay: none

### Refinement

**Table:**

Refinement on *F*^2^	Secondary atom site location: difference Fourier map
Least-squares matrix: full	Hydrogen site location: difference Fourier map
*R*[*F*^2^ > 2σ(*F*^2^)] = 0.035	All H-atom parameters refined
*wR*(*F*^2^) = 0.079	*w* = 1/[σ^2^(*F*_o_^2^) + (0.025*P*)^2^ + 0.41*P*] where *P* = (*F*_o_^2^ + 2*F*_c_^2^)/3
*S* = 1.01	(Δ/σ)_max_ = 0.003
2466 reflections	Δρ_max_ = 0.35 e Å^−^^3^
139 parameters	Δρ_min_ = −0.34 e Å^−^^3^
Primary atom site location: structure-invariant direct methods	Extinction correction: none

### Special details

**Table:**

Geometry. All e.s.d.'s (except the e.s.d. in the dihedral angle between two l.s. planes) are estimated using the full covariance matrix. The cell e.s.d.'s are taken into account individually in the estimation of e.s.d.'s in distances, angles and torsion angles; correlations between e.s.d.'s in cell parameters are only used when they are defined by crystal symmetry. An approximate (isotropic) treatment of cell e.s.d.'s is used for estimating e.s.d.'s involving l.s. planes.
Refinement. Refinement of *F*^2^ against ALL reflections. The weighted *R*-factor *wR* and goodness of fit *S* are based on *F*^2^, conventional *R*-factors *R* are based on *F*, with *F* set to zero for negative *F*^2^. The threshold expression of *F*^2^ > 2σ(*F*^2^) is used only for calculating *R*-factors(gt) *etc*. and is not relevant to the choice of reflections for refinement. *R*-factors based on *F*^2^ are statistically about twice as large as those based on *F*, and *R*- factors based on ALL data will be even larger.

### Fractional atomic coordinates and isotropic or equivalent isotropic displacement parameters (Å^2^)

**Table:**

	*x*	*y*	*z*	*U*_iso_*/*U*_eq_	
P1	0.10537 (7)	0.60187 (2)	0.89929 (7)	0.03542 (12)	
O1	0.0298 (2)	0.59213 (9)	0.6716 (2)	0.0477 (3)	
H1	0.114 (6)	0.606 (2)	0.586 (6)	0.145 (15)*	
O2	0.32968 (19)	0.59006 (8)	0.96702 (19)	0.0455 (3)	
C1	0.0385 (5)	0.68801 (12)	0.9733 (4)	0.0563 (6)	
H11	0.067 (4)	0.6933 (14)	1.116 (4)	0.070 (8)*	
H12	0.106 (5)	0.7222 (17)	0.917 (5)	0.094 (10)*	
H13	−0.088 (4)	0.6940 (15)	0.925 (4)	0.075 (9)*	
C2	−0.0220 (3)	0.53869 (9)	1.0246 (3)	0.0371 (4)	
H21	0.017 (3)	0.5445 (12)	1.163 (3)	0.047 (6)*	
H22	−0.157 (3)	0.5475 (12)	0.984 (3)	0.051 (6)*	
P2	0.48253 (7)	0.61724 (2)	0.51341 (7)	0.03302 (11)	
O3	0.25753 (19)	0.62778 (7)	0.44442 (18)	0.0412 (3)	
O4	0.5560 (2)	0.62903 (7)	0.7409 (2)	0.0421 (3)	
H4	0.470 (5)	0.6132 (18)	0.826 (5)	0.117 (12)*	
C3	0.6086 (4)	0.67852 (12)	0.3826 (4)	0.0501 (5)	
H31	0.575 (4)	0.7228 (16)	0.410 (4)	0.078 (8)*	
H32	0.565 (4)	0.6729 (14)	0.247 (4)	0.065 (8)*	
H33	0.738 (4)	0.6751 (15)	0.426 (4)	0.079 (9)*	
C4	0.5508 (3)	0.52908 (9)	0.4481 (3)	0.0343 (3)	
H41	0.693 (3)	0.5257 (11)	0.494 (3)	0.042 (5)*	
H42	0.513 (3)	0.5270 (11)	0.311 (3)	0.044 (6)*	

### Atomic displacement parameters (Å^2^)

**Table:**

	*U*^11^	*U*^22^	*U*^33^	*U*^12^	*U*^13^	*U*^23^
P1	0.0352 (2)	0.0375 (2)	0.0329 (2)	−0.00097 (17)	0.00569 (17)	0.00385 (18)
O1	0.0359 (7)	0.0712 (10)	0.0338 (7)	−0.0062 (6)	0.0024 (5)	0.0038 (6)
O2	0.0350 (7)	0.0632 (9)	0.0364 (7)	−0.0004 (6)	0.0030 (5)	0.0047 (6)
C1	0.0677 (16)	0.0365 (10)	0.0624 (16)	0.0033 (10)	0.0087 (12)	0.0018 (10)
C2	0.0403 (9)	0.0362 (9)	0.0361 (9)	0.0017 (7)	0.0108 (7)	0.0015 (7)
P2	0.0359 (2)	0.02862 (19)	0.0346 (2)	−0.00337 (16)	0.00759 (17)	0.00217 (17)
O3	0.0379 (7)	0.0472 (7)	0.0371 (7)	0.0038 (5)	0.0047 (5)	0.0049 (5)
O4	0.0374 (7)	0.0493 (8)	0.0378 (7)	−0.0064 (6)	0.0037 (5)	−0.0068 (6)
C3	0.0576 (14)	0.0369 (10)	0.0563 (13)	−0.0105 (9)	0.0133 (11)	0.0095 (9)
C4	0.0380 (9)	0.0299 (8)	0.0357 (9)	−0.0019 (7)	0.0095 (7)	0.0018 (7)

### Geometric parameters (Å, °)

**Table:**

P1—O2	1.5096 (14)	P2—O3	1.5115 (14)
P1—O1	1.5452 (15)	P2—O4	1.5468 (14)
P1—C1	1.776 (2)	P2—C3	1.779 (2)
P1—C2	1.7850 (19)	P2—C4	1.7942 (19)
O1—H1	0.94 (4)	O4—H4	0.95 (4)
C1—H11	0.96 (3)	C3—H31	0.89 (3)
C1—H12	0.92 (3)	C3—H32	0.92 (3)
C1—H13	0.86 (3)	C3—H33	0.87 (3)
C2—C2^i^	1.529 (3)	C4—C4^ii^	1.539 (3)
C2—H21	0.93 (2)	C4—H41	0.95 (2)
C2—H22	0.91 (2)	C4—H42	0.92 (2)
			
O2—P1—O1	113.08 (8)	O3—P2—O4	112.74 (8)
O2—P1—C1	110.24 (12)	O3—P2—C3	108.55 (11)
O1—P1—C1	110.11 (12)	O4—P2—C3	109.08 (11)
O2—P1—C2	108.23 (9)	O3—P2—C4	109.76 (8)
O1—P1—C2	108.33 (9)	O4—P2—C4	109.63 (8)
C1—P1—C2	106.60 (11)	C3—P2—C4	106.90 (11)
P1—O1—H1	119 (2)	P2—O4—H4	118 (2)
P1—C1—H11	112.0 (16)	P2—C3—H31	108.9 (18)
P1—C1—H12	109.5 (19)	P2—C3—H32	110.5 (16)
H11—C1—H12	110 (2)	H31—C3—H32	106 (2)
P1—C1—H13	107.7 (19)	P2—C3—H33	109.7 (19)
H11—C1—H13	110 (2)	H31—C3—H33	106 (3)
H12—C1—H13	107 (3)	H32—C3—H33	115 (2)
C2^i^—C2—P1	112.62 (17)	C4^ii^—C4—P2	111.78 (16)
C2^i^—C2—H21	107.8 (14)	C4^ii^—C4—H41	108.8 (12)
P1—C2—H21	110.3 (13)	P2—C4—H41	106.0 (12)
C2^i^—C2—H22	109.4 (15)	C4^ii^—C4—H42	112.7 (13)
P1—C2—H22	106.6 (14)	P2—C4—H42	105.1 (13)
H21—C2—H22	110.1 (18)	H41—C4—H42	112.3 (17)
			
O2—P1—C2—C2^i^	−61.3 (2)	O3—P2—C4—C4^ii^	−62.1 (2)
O1—P1—C2—C2^i^	61.7 (2)	O4—P2—C4—C4^ii^	62.3 (2)
C1—P1—C2—C2^i^	−179.8 (2)	C3—P2—C4—C4^ii^	−179.64 (19)

Symmetry codes: (i) −*x*, −*y*+1, −*z*+2; (ii) −*x*+1, −*y*+1, −*z*+1.

### Hydrogen-bond geometry (Å, °)

**Table:**

*D*—H···*A*	*D*—H	H···*A*	*D*···*A*	*D*—H···*A*
O4—H4···O2	0.95 (4)	1.55 (4)	2.504 (2)	178 (3)
O1—H1···O3	0.94 (4)	1.56 (4)	2.499 (2)	179 (4)
